# Multi-locus sequence typing of African swine fever viruses from endemic regions of Kenya and Eastern Uganda (2011–2013) reveals rapid *B602L* central variable region evolution

**DOI:** 10.1007/s11262-017-1521-4

**Published:** 2017-11-15

**Authors:** C. K. Onzere, A. D. Bastos, E. A. Okoth, J. K. Lichoti, E. N. Bochere, M. G. Owido, G. Ndambuki, M. Bronsvoort, R. P. Bishop

**Affiliations:** 1 International Livestock Research Institute (ILRI), PO Box 30709, Nairobi, 00100 Kenya; 2 Department of Zoology and Entomology, Mammal Research Institute, University of Pretoria, Private Bag 20, Hatfield, 0028 South Africa; 3 State Department of Veterinary Services, Ministry of Agriculture, Livestock and Fisheries, Private Bag-00625, Nairobi, Kenya; 4 College of Medicine and Veterinary Medicine, University of Edinburgh, The Chancellor’s Building, 49 Little France Crescent, Edinburgh, EH16 4S Scotland, UK

**Keywords:** African swine fever virus, Genotype IX, East Africa, CVR, Thymidine kinase

## Abstract

The central variable region (CVR) within the *B602L* gene of the African swine fever virus (ASFV) is highly polymorphic within the 23 ASFV genotypes defined by sequencing of the C-terminal end of the *p72* locus. Sequencing the *p54* gene further discriminates ASFV genotypes that are conserved at the *p72* locus. Variation in the thymidine kinase locus is a novel additional tool for ASFV genotyping whose application for this purpose is described for the first time herein. We evaluated genetic variation at these four polymorphic loci in 39 ASFV isolates obtained from outbreaks in Kenya and a region of Eastern Uganda between 2011 and 2013. Analysis of the *p72* and *p54* loci revealed high genetic conservation among these isolates; all clustered within *p72* genotype IX and were similar to isolates associated with earlier outbreaks in East Africa. The thymidine kinase gene of the Kenyan isolates in this study were distinct relative to Southern African isolates and synonymous substitutions were observed among viruses from central Kenya. Analysis of the CVR within the *B602L* gene revealed two previously unknown polymorphisms that were restricted to Western Kenya and Eastern Uganda. A novel variant was revealed within CVR subgroup XXIV and a novel CVR subgroup XXIVa that contains tetrameric repeat F which has previously only been associated with *p72* genotype I, was also identified for the first time in East Africa. Phylogeographic analysis of isolates based on CVR polymorphisms revealed rapid evolution and dissemination of variants present within ASFV genotype IX in East Africa.

**Electronic supplementary material:**

The online version of this article (10.1007/s11262-017-1521-4) contains supplementary material, which is available to authorized users.

## Introduction

African swine fever (ASF) is a highly contagious and frequently fatal hemorrhagic disease of swine that typically results in high mortality in domestic pigs and European wild boar. The disease is caused by the African swine fever virus (ASFV); a large virus that replicates within the cell’s cytoplasm and is the sole member of the family Asfarviridae, genus Asfivirus [1] and the only currently known DNA arbovirus. The ASFV genome consists of a single linear double stranded DNA molecule that is between 170 and 190 kilobase pairs in size [2] and depending on the isolate, it encodes between 151 and 167 open reading frames [3, 4].

The epidemiology of ASF is complex and differs across countries, regions, and continents depending on the presence or absence of wild suids, arthropod vectors, and pig production systems [5]. Transmission occurs through three distinct cycles, specifically an ancestral sylvatic cycle involving African wild suids and soft ticks (genus *Ornithodoros*), a domestic pig to pig cycle, which is probably the most frequent current mode of transmission in the majority of African pig production systems, and transmission between domestic pigs via *Ornithodoros* ticks, which has rarely been confirmed [6].

ASF has a devastating socio-economic impact both in endemic areas, where investment in the pig sector is reduced due to the risk of sporadic outbreaks and in newly infected regions [5] due to high mortality rates particularly in naïve populations. The potential threat to global food security and decreased investment in the pig industry represents a serious problem [7], given the history of this virus ‘escaping’ from the African continent. The transboundary nature of the threat posed by ASFV was emphasized by the incursion of ASFV genotype II into Georgia in 2007, and subsequently Russia [8] and most recently Eastern Europe with serious consequences for the global pig industry [9].

ASFV is endemic in East Africa and sporadic outbreaks are frequently reported in Kenya and Uganda. The virus was also recently detected for the first time in Ethiopia [10]. Despite this, the popularity of pig products in East Africa, in both Kenya, and particularly Uganda, is rising due to the fact that pork is a cheaper source of high quality protein as compared to beef. This has resulted in the rapid growth of the smallholder pig industry in these countries providing a source of income generation for resource-poor, small-scale farmers. Recent regular ASF outbreaks therefore represent a significant economic constraint to smallholders [6, 11].

Studies conducted by Gallardo and colleagues have highlighted the likelihood of transboundary transmission of ASFV in East Africa especially between Kenya and Uganda in either direction [12, 13]. Molecular analysis indicated that the 2006–2007 outbreaks in Kenya were caused by a virus that is genetically very similar to a virus isolated from Uganda within the same period [13]. ASFV genotype IX is known to be highly virulent and causes extremely high mortality in naive pigs. It has been associated with sporadic but persistent ASF outbreaks in both free range and housed small-scale Kenyan pig farms between 2005 and 2011 [12–14].

In order to investigate the molecular epidemiology of ASFV associated with outbreaks in Kenya and Eastern Uganda between 2011 and 2013, comprehensive genotypic characterization of four polymorphic loci was conducted. This encompassed: genotyping of the variable 3′-end of the *B646L* gene that encodes the major capsid protein *p72* in order to classify the genotype responsible for the outbreaks within the 23 published *p72*-based ASFV genotypes [10, 14]; The *E183L* gene that encodes the *p54* ASFV protein was examined for potentially enhanced resolution of the genotype(s) identified by sequencing the *B646L* gene [12]; The thymidine kinase (*TK*) gene that is essential in ASFV DNA synthesis was also analyzed [15]; Fourthly, the *B602L* gene that encodes a chaperone which contains a central variable region (CVR) comprising tetrameric repeats [16] was also sequenced in order to maximize discrimination of isolates that were identical within the *p72*, *p54,* and *TK* genes. Analysis of the CVR of the *B602L* gene facilitated subtyping of the major ASFV genotypes obtained by sequencing of the *p72* and *p54* genes [12] and variations within this locus were used to evaluate the transmission patterns of the virus at high resolution, both spatially and temporally providing increased insight into the epidemiology and dissemination of novel viral variants in Kenya.

## Materials and methods

### Study areas and sampling procedure

**Fig. 1:**
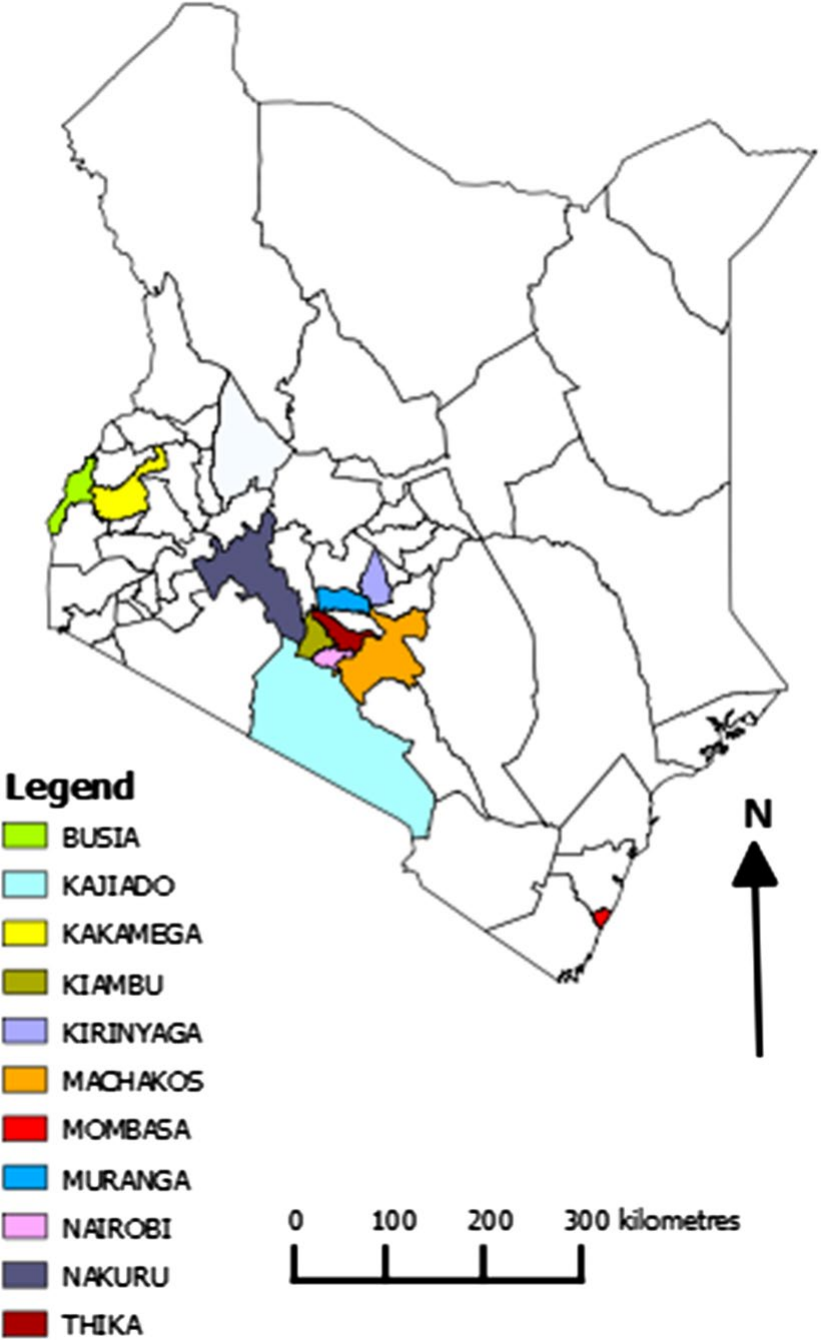
Map of Kenya showing reported outbreak areas in Kenya between September 2011 and December 2013

Three sampling strategies were utilized in obtaining samples for subsequent ASFV diagnosis and genotyping. The first involved sampling domestic pigs from reported outbreak areas across Kenya was between September 2011 and December 2013 (Fig. 1); a total of 110 pigs were sampled by the Ministry of Livestock through the Department of Veterinary services (DVS)—Kenya. The samples collected comprised 233 tissue samples, 24 blood samples, and 13 serum samples. These outbreak samples were sent to the International Livestock Research Institute (ILRI) under a cold chain to enable diagnostic analysis for the presence of ASFV.

The second strategy entailed sampling of domestic pigs in ASFV endemic districts within Busia County at the Kenya–Uganda border, specifically Busia and Teso districts. This area was chosen due to the likelihood of detection of transboundary virus transfer that was implied through data obtained in earlier studies [12]. In this case, a systematic sampling approach was utilized in the selection of 320 households [14]. Sampling was also performed in the neighboring districts of Eastern Uganda (Busia and Tororo) when outbreaks were reported in Busia and Teso districts. A total of 817 blood samples, 817 serum samples and 14 tissue samples were collected for analysis.

The third strategy involved sampling of 40 domestic pigs in 2013 from abattoirs in Busia County. The animals were examined for symptoms of ASF before slaughter and post-mortems were performed subsequently by ILRI and DVS veterinarians; a total of 40 blood, 40 serum, and 161 tissue samples were obtained to further investigate the presence or absence of ASFV using nucleic acid and serological diagnostics.

In all the three sampling strategies, blood samples were collected in BD Vacutainer^®^ 10 ml EDTA tubes and serum samples were collected in serum BD Vacutainer^®^ 10 ml tubes. In cases where the animals had died, various tissue samples were collected from each animal during the post-mortem analysis and placed in sterile BD 50 ml falcon^®^ tubes without preservatives. All samples collected were maintained under a strict − 80 °C cold chain.

### DNA isolation and ASFV DNA detection

#### Tissue sample processing

Each tissue sample was thawed at room temperature; a small portion of the tissue was excised using a sterile surgical blade and weighed. 1.5 mg of the tissue was placed in a labeled sterile 1.5 ml eppendorf tube. The tissue was then crushed in liquid nitrogen using a pestle and mortar and suspended in 500 µl of PBS pH 7.0. The mixture was thoroughly vortexed then centrifuged at 13,000 RPM in a microcentrifuge for 10 min. The supernatant was slowly pipetted out and dispensed into a sterile and clearly labeled 1.5 ml eppendorf tube.

#### DNA extraction

DNA was extracted from 200 µl of each blood sample, serum sample, and tissue lysate using the DNeasy Blood and Tissue kit (Qiagen, ref # 69506) according to the manufacturer’s instructions. Positive (PEC) and negative extraction (NEC) controls were included to ensure that the process was successful and to check for contamination, respectively. The PEC consisted of a known ASFV positive blood sample whilst the NEC consisted of sterile phosphate-buffered saline (PBS) pH 7.0. DNA quantification of each sample was determined using a Nanodrop 1000 spectrophotometer (Thermoscientific).

#### ASFV diagnosis

Two diagnostic assays were utilized for the detection of ASFV in the extracted DNA samples. A ‘hot-start’ gel-based PCR assay, using the PPA1/PPA2 *p72* gene primer set that targets a 257 bp amplicon, was initially used to detect the presence of ASFV, as recommended by the OIE [17]. All DNA extracts were then subjected to a Universal probe library (UPL) real-time PCR [18] to confirm the primary PCR results. The secondary UPL PCR assay permitted detection of virus in samples with low infection levels, not detectable using the conventional PCR assay. Samples with cycle threshold (Ct) values less than 40 were considered positive, while those with Ct values > 40 were regarded as inconclusive/ambiguous.

### ASFV genomic characterization

Genetic analysis of ASFV was conducted using PCR amplification of the four polymorphic loci with primer sets unique to the specific loci. These comprised: (a) the *p72*-derived *p72*U/*P72*D primers in the amplification of a 478 bp region within the *B646L/p72* gene [19]; (b) The PPA89/PPA722 primers which amplify a 676 bp region within the *E183L/p54* gene [12]; (c) The *TK*1 (CGC GTC TTA CTA AAA GTG A) and *TK*-Rev (TAG CAG AGT AAT AAA CTC TT) primers utilized in the amplification of a 750 bp region within the thymidine kinase gene; and (d) the CVR1/CVR2 primer set in the amplification of fragments of variable size (up to 665 bp) within the *B602L* gene [16].

The discrete bands obtained from the amplification of each locus were purified from the agarose gels using the QIAquick gel extraction kit (Qiagen, ref # 28706) according to the manufacturer’s instructions. The purified products were then directly sequenced by the Sanger method.

The primary sequence data were analyzed using BIOEDIT and MEGA version 6.06 to perform multiple alignments in comparison to reference sequences corresponding to the four polymorphic loci obtained from GenBank. Translation of the nucleotide sequences to amino acid sequences for the thymidine kinase and *B602L* genes was performed using the RevTrans 2.0 software [20]. Alignment of the amino acid sequences was then effected using the Sequence Alignment publishing Tool (SeqPublish) available in the HIV sequence database with the aim of identifying variants [21]. ElimDupes also available in the HIV sequence database was used to eliminate duplicate sequences in order to identify unique sequences [21].

### Phylogenetic analysis

Phylogenetic analysis of each locus was performed using MEGA version 6.06 and phylogenetic trees were constructed using a Minimum Evolution algorithm following initial tree construction using the neighbor joining method. The P-distance and a defined nucleotide substitution model were also utilized in the construction of the phylogenetic trees. Additionally, the data from each locus were re-sampled 1000 times using the bootstrap method [22].

Bayesian Evolutionary Analysis by Sampling Trees (BEAST) was used to estimate the origin of ASFV variants with reference to the sequence data obtained from the CVR of the *B602L* ORF. BEAST was used following initial application of the Bayesian Evolutionary Analysis Utility (BEAUti) [23]. The output obtained from BEAST was further analyzed using the Spatial Phylogenetic Reconstruction of EvolutionAry Dynamics (SPREAD), which is key to the implementation of phylogeographic analysis using BEAST. To enable visualization of inferred epidemiological relationships of the African swine fever virus in Kenya, the output from SPREAD was loaded onto Google Earth [24].

## Results

### Detection of ASFV DNA by PCR

A total of 39 domestic pigs tested ASFV positive by both conventional and UPL PCR and an additional 2 pigs were confirmed to be positive by UPL PCR (Table 1). The table also links the diagnostic results to the origin of the samples collected between September 2011 and December 2013. Only the 39 pigs that tested positive on both conventional and UPL PCR (Ct < 30) were selected for sequencing and subsequent genotyping.

**Table 1:** Summary of diagnostic results obtained using both the hot-start gel-based PCR and the UPL real-time PCR assay across the three sampling strategies between 2011 and 2013

Year	Month	Reason for sampling	Sampling location	Number of domestic pigs sampled	ASFV DNA detection results		Genotyped
			(Town/District)		Conventional PCR	UPL PCR	
					No. of positive pigs	No. of positive pigs	
2011	Sep	Suspected outbreak	Kisauni	2	2	2	*
	Dec	Suspected outbreak	Nairobi	2	0	0	
		Suspected outbreak	Kiambu	2	2	2	*
2012	Jan	Suspected outbreak	Kiambu	7	1	1	*
	Feb	Suspected outbreak	Kiambu	1	0	0	
		Suspected outbreak	Nyeri	1	0	1	
		Suspected outbreak	Machakos	1	1	1	*
	Mar	Suspected outbreak	Athi river	2	2	2	*
		Suspected outbreak	Thika	1	1	1	
		Suspected outbreak	Kikuyu	1	1	1	
	Apr	Suspected outbreak	Nakuru	1	1	1	
	May	Suspected outbreak	Kiambu	2	1	1	*
	Jun	Suspected outbreak	Nairobi	3	3	3	*
		Suspected outbreak	Busia (Burumba)	5	1	1	*
		Suspected outbreak	Kiambu	2	1	1	*
		Suspected outbreak	Kajiado	1	1	1	
	Jul	Suspected outbreak	Kiambu	2	0	0	
		Suspected outbreak	Nairobi	1	0	0	
	Aug	Suspected outbreak	Kiambu	2	1	1	
	Sep	Suspected outbreak	Kiambu	21	1	1	*
	Oct	Suspected outbreak	Kajiado	2	3	3	
		Suspected outbreak	Kiambu	1	0	0	
		Suspected outbreak	Limuru	1	1	1	
		Suspected outbreak	Busia (Sigalame)	1	1	1	*
	Nov	Suspected outbreak	Busia (Ameri)	1	1	1	
	Dec	Suspected outbreak	Nairobi	1	0	0	
		Suspected outbreak	Uasin Gishu	8	0	1	
	Dec	Suspected outbreak	Muranga	1	1	1	*
	July–October	Endemic ASF districts	Teso and Busia Districts	605	0	0	
2013	Jan	Endemic ASF districts	Teso, Busia, & Eastern Uganda	125	2	2	*
	Apr	Suspected outbreak	Kiambu	2	1	1	*
		Suspected outbreak	Kirinyaga	2	0	0	
	May	Abattoirs	Busia	24	0	0	
	Jun	Suspected outbreak	Machakos	1	1	1	
		Suspected outbreak	Muranga	1	1	1	
		Suspected outbreak	Kiambu	1	0	0	
	Jul	Endemic ASF districts	Teso, Busia, & Eastern Uganda	87	4	4	*
		Suspected outbreak	Kiambu	1	1	1	*
		Suspected outbreak	Kirinyaga	2	1	1	*
	Aug	Suspected outbreak	Nakuru	3	1	1	*
		Suspected outbreak	Kiambu	2	1	1	*
	Sep	Suspected outbreak	Nyadorera (Nyanza)	1	1	1	*
		Suspected outbreak	Kiambu	3	1	1	*
		Suspected outbreak	Nairobi	1	1	1	
	Oct	Suspected outbreak	Busia	1	1	1	*
		Suspected outbreak	Kajiado	2	0	0	
		Suspected outbreak	Kiambu	1	0	0	
		Abattoirs	Busia	16	5	5	*
	Nov	Suspected outbreak	Kiambu	4	1	1	*
	Dec	Suspected outbreak	Kakamega	5	1	1	*
		Suspected outbreak	Kiambu	1	1	1	*

Only three suspected outbreaks were reported between September 2011 (when the study began) and December 2011. Domestic pigs selected for genotyping are indicated by *

### Genotypic characterization

#### Phylogenetic analysis of the *B646L*/*p72* gene

Analysis of the *B646L* gene that encodes the C-terminal end of the *p72* major surface protein involved nucleotide sequences obtained from the 39 ASFV positive isolates sampled between 2011 and 2013 in Kenya and a small region of Eastern Uganda. The isolates were compared to 53 ASFV *p72* sequences retrieved from GenBank. It was evident that all the Kenyan isolates analyzed in this study were 100% identical at the nucleotide level and they clustered within *p72* genotype IX as indicated in supplementary Fig. 1.

#### Phylogenetic analyses of the *E183L/p54* gene

Sequence analysis of the *E183L* gene of the 39 ASFV isolates analyzed in this study also revealed high genetic conservation between the isolates. The nucleotide sequences were identical to those isolated in earlier outbreaks within East Africa [12, 24]. Thus the 2011 to 2013 ASFV isolates analyzed in this study not only clustered within genotype IX based on analysis of the *B646L* gene, but were also identical within the *E183L* gene (Supplementary Fig. 2).

**Fig. 2:**
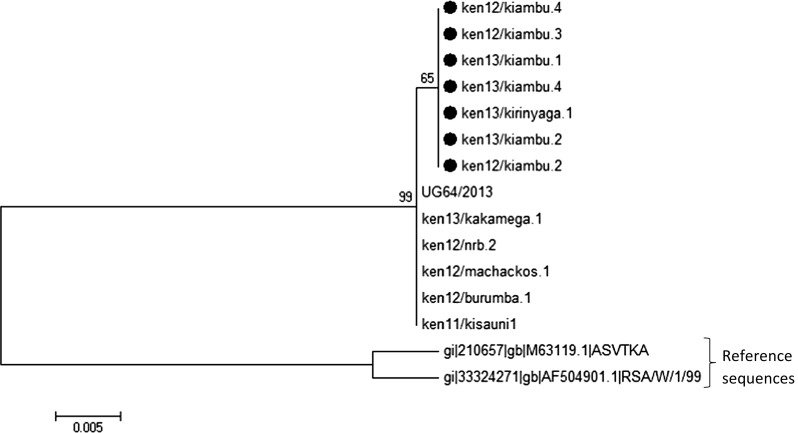
Phylogenetic tree highlighting the variations within the 2011 to 2013 ASFV Kenyan isolates due to nucleotide substitutions in the thymidine kinase gene within isolates obtained from Central Kenya that are indicated by ●

#### Phylogenetic analysis using the thymidine kinase gene

**Fig. 3:**
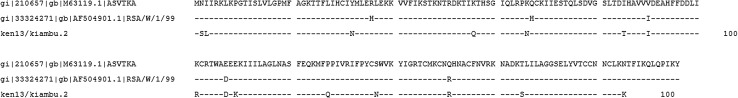
Thymidine kinase amino acid sequences translated using SeqPublish highlighting ken13/kiambu.2 (as a representative of 13 ASFV Kenyan isolates) in comparison to South African reference sequences obtained from GenBank. ken13/kiambu.2 was identical to ken13/kiambu.1, ken13/kiambu.4, ken12/kiambu.2, ken12/kiambu.3, ken12/Kiambu.4, ken11/kisauni1, ken13/kirinyaga.1, ken12/burumba.1, ken12/machakos.1, ken12/nrb.2, and ken13/kakamega.1

Of the 39 ASFV positive isolates, only 14 gave high quality reads upon sequencing of the thymidine kinase (*TK*) gene and thus were selected for comparative analysis with sequences available in GenBank. The 14 ASFV isolates were highly similar in sequence at the nucleotide level with a limited number of nucleotide substitutions observed between the Central Kenya isolates (Fig. 2). It was evident that the substitutions were synonymous following the application of RevTrans and SeqPublish in the analysis of the corresponding amino acid sequences (Supplementary Fig. 3). However, the 2011–2013 isolates analyzed in this study were typically distinct from the South African reference sequences obtained from GenBank (Fig. 3) suggesting that the *TK* gene may be an additional tool for differentiating isolates from different regions.

#### Analysis of the central variable region (CVR) within the B602L gene open reading frame

**Fig. 4:**
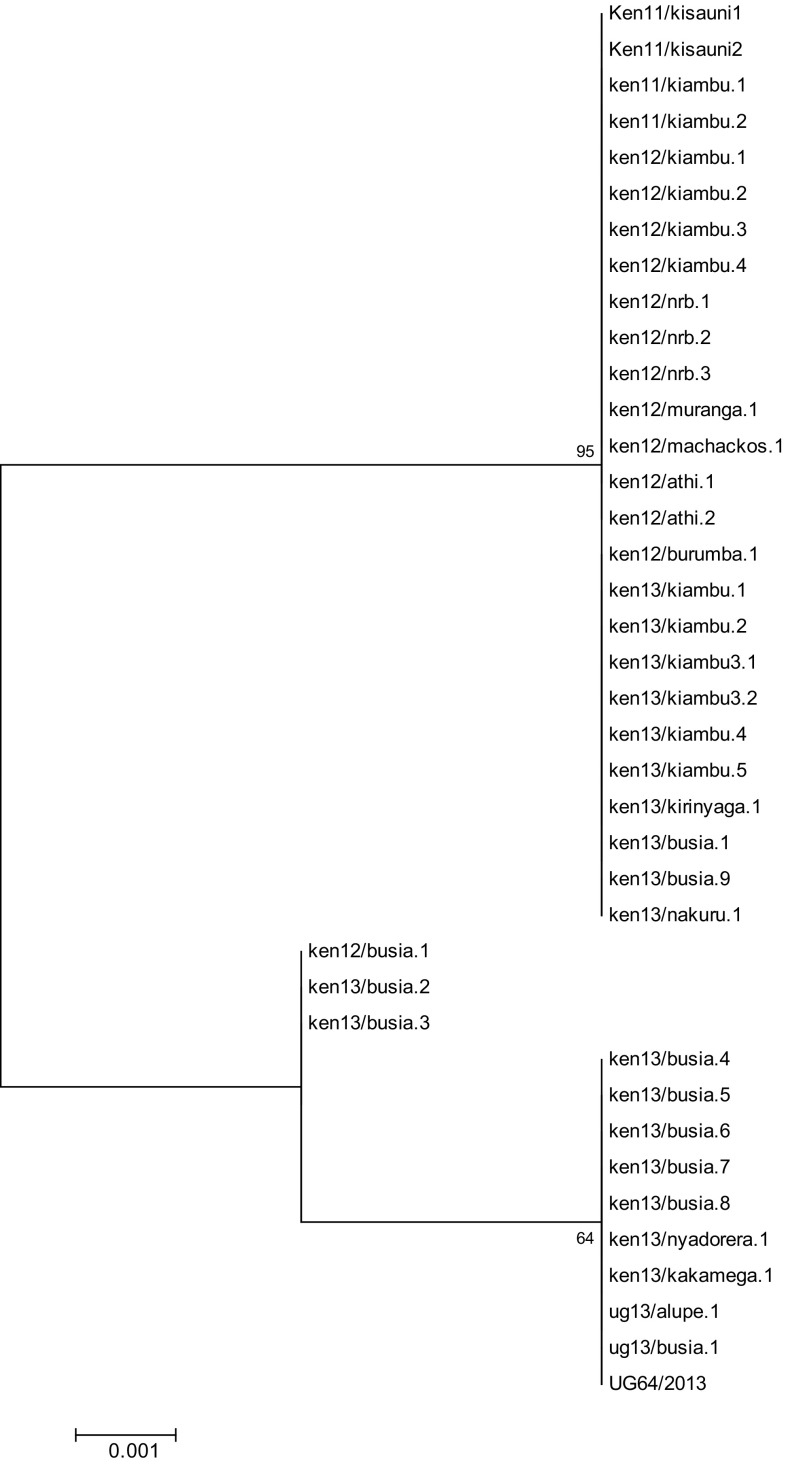
Phylogenetic tree derived from the *B602L* ORF showing the 2011 to 2013 Kenyan and Eastern Uganda ASFV isolates. The tree illustrates three clusters identified by nucleotide polymorphisms between positions 185 and 189 of the nucleotide alignment defined by reference to annotated sequences available in GenBank

All the 39 *p72* genotype IX isolates characterized in this study clustered within a CVR allele type characterized by an insertion of twelve nucleotides from positions 201 to 212 of the nucleotide alignment [13]. However, novel nucleotide polymorphisms characterized by substitutions of three or four nucleotides located between positions 185 and 189 of the nucleotide alignment were also observed when compared to reference sequences obtained from GenBank and this led to division of the isolates analyzed in this study into three different clusters as illustrated in Fig. 4. Additional substitutions identical to Ugandan isolates sampled between 2010 and 2013 [25] were noted in positions 317 and 318 of the nucleotide alignment in which nucleotides CA were substituted by TG and nucleotide T at position 333 was substituted by C in all the ASFV isolates analyzed in this study.

The CVR nucleotide sequences were translated using the RevTrans 2.0 software and the amino acid sequences aligned using SeqPublish; the output is illustrated in Table 2. ElimDupes was then used to eliminate duplicate sequences and finalize the output. This analysis revealed the presence of three unique sequences representing non-synonymous substitutions created by the nucleotide variations within the *B602L* ORF between positions 185 and 189 of the nucleotide alignment. The unique sequences occurred as a result of three amino acid pairs i.e., AD, AN, and VS that correspond to three different tetrameric amino acid repeats within the same region i.e., C**AD**T, C**AN**T, and C**VS**T with reference to the previously described classification system [16]. C**AD**T and C**AN**T represented novel tetrameric repeats within this region.

**Table 2:**
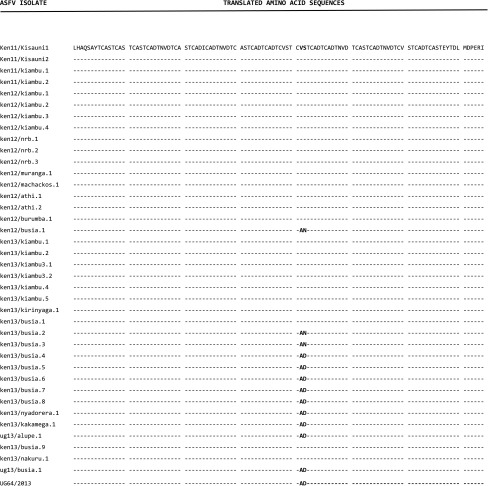
Translated amino acid sequences generated using SeqPublish highlighting the three variants within the CVR locus in the Kenyan and Eastern Uganda ASFV isolates collected between 2011 and 2013

C**VS**T was observed in 66.6% of the 2011–2013 isolates; this variant is also present in Kenyan and Ugandan ASFV isolates analyzed in earlier studies [13, 25]. C**AD**T was observed in 25.6% of the isolates and appears to be geographically restricted to areas within Western Kenya and Eastern Uganda in 2013 specifically Busia County and Kakamega County in Kenya and Alupe in Uganda that shares a border with Busia in Kenya. The C**AN**T variant exhibited the lowest frequency at 7.8% and was restricted to Busia County isolates collected in 2012 and 2013.

The additional substitutions within the CVR at positions 317, 318, and 333 of the nucleotide alignment yielded synonymous substitutions upon translation and thus had no effect on the amino acid composition of the locus.

The translated amino acid sequences resulted in the identification of 23 amino acid tetramers within the *B602L* ORF across all the ASFV isolates analyzed in this study following application of the original coding system [16] as summarized in Table 3. The amino acid tetrameric repeats included AAABNABBNABBaFBBNABNaBA and AAABNABBNABBaBBBNABNaBA which corresponded to the novel variants CANT and CADT and clustered within CVR subgroups XXIVa and XXIV, respectively. The third variant i.e., AAABNABBNABBAABBNABNABA was categorized within the CVR subgroup XXIV and was identical to Kenyan and Ugandan isolates analyzed in earlier studies performed between 2003 and 2013 [12, 13, 24].

**Table 3:** Amino acid sequence alignment of the tetrameric repeats that constitute the central variable region of the *B602L* ORF within the 2011–2013 Kenyan and Eastern Ugandan isolates belonging to *p72* genotype IX

ASFV isolate	Tetrameric repeats	Number of repeats	CVR subgroup
Uga 95/1 (reference)	AAABNABBNABBNABAABBNABNABA	26	XXIV
Ken11/Kisauni1	AAABNABBNAB - - - BAABBNABNABA	23	XXIV
Ken11/Kisauni2	AAABNABBNAB - - - BAABBNABNABA	23	XXIV
ken11/kiambu.1	AAABNABBNAB - - - BAABBNABNABA	23	XXIV
ken11/kiambu.2	AAABNABBNAB - - - BAABBNABNABA	23	XXIV
ken12/kiambu.1	AAABNABBNAB - - - BAABBNABNABA	23	XXIV
ken12/kiambu.2	AAABNABBNAB - - - BAABBNABNABA	23	XXIV
ken12/kiambu.3	AAABNABBNAB - - - BAABBNABNABA	23	XXIV
ken12/kiambu.4	AAABNABBNAB - - - BAABBNABNABA	23	XXIV
ken12/nrb.2	AAABNABBNAB - - - BAABBNABNABA	23	XXIV
ken12/nrb.3	AAABNABBNAB - - - BAABBNABNABA	23	XXIV
ken12/muranga.1	AAABNABBNAB - - - BAABBNABNABA	23	XXIV
ken12/machackos.1	AAABNABBNAB - - - BAABBNABNABA	23	XXIV
ken12/athi.1	AAABNABBNAB - - - BAABBNABNABA	23	XXIV
ken12/athi.2	AAABNABBNAB - - - BAABBNABNABA	23	XXIV
ken12/burumba.1	AAABNABBNAB - - - BAABBNABNABA	23	XXIV
ken12/busia.1	AAABNABBNAB - - - BAFBBNABNABA	23	XXIVa
ken13/kiambu.1	AAABNABBNAB - - - BAABBNABNABA	23	XXIV
ken13/kiambu.2	AAABNABBNAB - - - BAABBNABNABA	23	XXIV
ken13/kiambu3.1	AAABNABBNAB - - - BAABBNABNABA	23	XXIV
ken13/kiambu3.2	AAABNABBNAB - - - BAABBNABNABA	23	XXIV
ken13/kiambu.4	AAABNABBNAB - - - BAABBNABNABA	23	XXIV
ken13/kiambu.5	AAABNABBNAB - - - BAABBNABNABA	23	XXIV
ken13/kirinyaga.1	AAABNABBNAB - - - BAABBNABNABA	23	XXIV
ken13/busia.1	AAABNABBNAB - - - BAABBNABNABA	23	XXIV
ken13/busia.2	AAABNABBNAB - - - BAFBBNABNABA	23	XXIVa
ken13/busia.3	AAABNABBNAB - - - BAFBBNABNABA	23	XXIVa
ken13/busia.4	AAABNABBNAB - - - BABBBNABNABA	23	XXIV
ken13/busia.5	AAABNABBNAB - - - BABBBNABNABA	23	XXIV
ken13/busia.6	AAABNABBNAB - - - BABBBNABNABA	23	XXIV
ken13/busia.7	AAABNABBNAB - - - BABBBNABNABA	23	XXIV
ken13/busia.8	AAABNABBNAB - - - BABBBNABNABA	23	XXIV
ken13/nyadorera.1	AAABNABBNAB - - - BABBBNABNABA	23	XXIV
ken13/kakamega.1	AAABNABBNAB - - - BABBBNABNABA	23	XXIV
ug13/alupe.1	AAABNABBNAB - - - BABBBNABNABA	23	XXIV
ken13/busia.9	AAABNABBNAB - - - BAABBNABNABA	23	XXIV
ken13/nakuru.1	AAABNABBNAB - - - BAABBNABNABA	23	XXIV
ug13/busia.1	AAABNABBNAB - - - BABBBNABNABA	23	XXIV
UG64/2013	AAABNABBNAB - - - BABBBNABNABA	23	XXIV

Single letter codes substituted for the tetrameric repeats are indicated as: A (CAST); a (CVST); B (CADT, CADI); N (NVDT); F (CANT). Dashes have been introduced manually to facilitate ease in visualization of similarities between the sequences

The genotypic sequences obtained from ASFV isolates analyzed in this study were deposited in GenBank and the accession numbers have been summarized in supplementary Table 1.

### Phylogeographic analysis of the central variable region

The *B602L* gene exhibits very frequent and rapid variation due to divergence of tetramer sequences, probably as a result of unequal crossing over or slippage during replication resulting in the derivation of novel isolates [16]. A Bayesian phylogeographic approach was used to estimate the ancestral locations of the isolates and the most significant epidemiological associations were inferred using the Bayesian Stochastic Search Variable Selection (BSSVS) procedure.

**Fig. 5:**
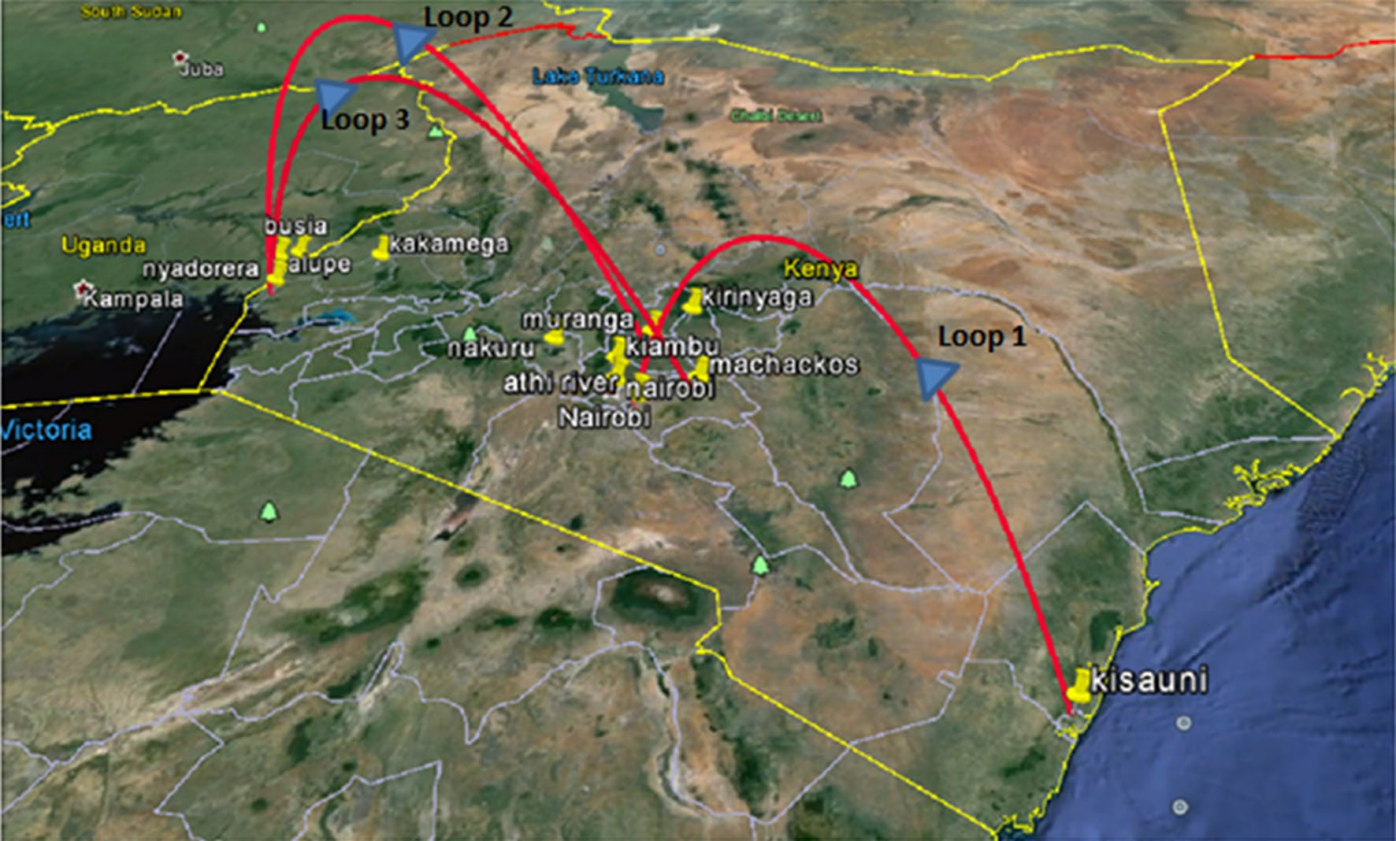
BEAST analysis displayed within a Google Earth map illustrating inferred transmission patterns of the African swine fever virus isolates associated with outbreaks between 2011 and 2013 in Kenya based on *B602L* CVR amino acid sequences. Loops 1 to 3 highlight the predicted directions of ASFV transmission

This involved initial uploading of the CVR sequences as a nexus file onto BEAUTi which was the key step in the initial establishment of the evolutionary model and Markov Chain Monte Carlo (MCMC) analysis options. The XML output from BEAUTi was then uploaded onto BEAST version 1.6.1. The log files obtained from BEAST and locations data were uploaded onto SPREAD enabling the phylogeographic visualization of inferred ASFV transmission within Kenya based on the CVR sequences of the isolates collected between 2011 and 2013. The KML output from SPREAD was uploaded onto Google Earth for an interactive visualization of the ASFV transmission patterns as shown in Fig. 5.

## Discussion

This study confirmed the endemic status of African swine fever virus in Western Kenya and Eastern Uganda and that the majority of outbreaks in the region occur as a result of ASFV genotype IX. The data strongly suggest that one major route of spread of the virus is along the Mombasa–Busia highway that links the major port city of Mombasa in Kenya to landlocked countries in East Africa, particularly Uganda which has a rapidly growing pig industry. The probable linkage of virus spread to transport and trade of infected domestic pigs and pork products along this route is highlighted by the molecular data.

Comparison of the three sampling strategies used in evaluating virus prevalence confirmed that sampling during suspected outbreaks provided an opportunity for in-depth molecular analyses of the viruses that are associated with clinical disease in domestic pigs. By contrast the study also revealed that conducting epidemiological studies based on PCR assays in ASFV endemic areas with the goal of evaluating the prevalence of the virus was problematical in East Africa using currently available serological and nucleic acid-based techniques on blood, one likely factor being sequestration of the virus in tissues, resulting in underestimation of prevalence by sampling blood alone [14]. According to this study, only 0.83% of the domestic pigs sampled in the endemic districts tested ASFV positive using both PCR assays and these were sampled during secondary visits to farms, during the longitudinal phase of the study. This finding highlighted the possibility that ASFV frequently evades detection either due to low viral titers in blood that are below the detection threshold of the PCR assays, or presence of the virus in tissues that are difficult to sample routinely. Published data suggest that the apparently very low detection levels of ASFV in pigs in endemic areas could be due to the fact that once an outbreak occurs, most if not all domestic pigs in our study areas either succumb, or are sold, very rapidly to butchers or pig farmers from other communities [26, 27]. Farmers restock subsequently with ASFV naïve pigs. It is also probable that farmers quickly sell off their pigs, perhaps even before severe clinical symptoms are apparent due to lack of incentives to report to veterinary authorities and the mandatory slaughter and quarantine policies that are currently applied in the affected areas. This strategy allows farmers to circumvent losses during suspected outbreaks. These anthropogenic factors limit the accuracy of epidemiological studies of disease transmission in countries where ASFV occurs including the regions of Kenya and Uganda where our study was performed [27]. This almost certainly results in the underestimation of the level of circulating virus.

Sampling of domestic pigs in abattoirs prior to slaughter in Busia County revealed sequestration of the virus in tissues in 7.5% of the domestic pigs sampled. Interestingly, these animals appeared asymptomatic and were PCR negative when blood and serum samples were analyzed. The findings confirmed that the identification of ASFV genotypes IX and X in East Africa by PCR is frequently not possible in blood and serum [13]. Moreover, it is possible that ASFV remains dormant in tissues and subsequently reactivates because of biotic or abiotic stress resulting in sporadic outbreaks. However, it is also true that the detection of viral genomic DNA in tissues does not necessarily imply the presence of infectious virus particles.

The presence of infected pigs in abattoirs was consistent with the hypothesis that farmers sell off their pigs during suspected outbreaks and helps to explain the spread of ASFV through sale of pork products through informal markets or dissemination of pig waste in conjunction with poor/non-existent biosecurity practices [27]. This likely contributes to observed transmission pattern of the disease within Busia County and along the Mombasa–Busia highway.

Sequence analysis of the C-terminal end of the major capsid protein *p72* revealed high genetic conservation in all the isolates obtained from suspected outbreaks in Kenya and the small region in Eastern Uganda between 2011 and 2013; all the sequences were identical and they clustered within ASFV genotype IX [19]. The isolates were 100% identical to ASFV isolates associated with outbreaks between 2003 and 2007 in Kenya and Uganda [12] and more recent isolates analyzed in Uganda from 2010 to 2013 [28].

Analysis of the 2011–2013 ASFV isolates using the *E183L* gene that encodes the *p54* protein confirmed that the isolates not only clustered within *p72* genotype IX but also revealed high genetic conservation within *p54*. The thymidine kinase gene in the genotype IX isolates analyzed in this study were extremely distinct when compared to genotypes from southern Africa that had previously been sequenced and deposited in GenBank. The synonymous nature of the substitutions observed between isolates from central Kenya indicates that these mutations had no effect on the virus in terms of the amino acid composition of the protein and thus presumably no direct primary sequence effect on virus virulence. However, it is possible that the level of translation and hence protein expression levels could have been affected. This hypothesis will require further analysis.

Analysis of the CVR locus to facilitate sub-grouping of the 2011–2013 ASFV isolates revealed that all the isolates were characterized by 23 amino acid tetrameric repeats unlike most of the 2005–2008 Kenyan isolates [13] that were defined by 22 amino acid tetrameric repeats. The isolates analyzed in this study were genetically closer to the 2010 Kenyan isolates and the 2010–2013 Ugandan isolates [28] in respect of the number of tetrameric repeats. The dynamics of the tetrameric repeat number in isolates analyzed from outbreaks within Kenya and adjacent regions of Eastern Uganda highlights the fact that very rapid evolution can occur at the CVR within the *B602L* ORF, and is consistent with a previous report from Nigeria [29]. Further investigation is required to determine the cause of this rapid evolution for instance by examining the likelihood of inter-species transmission between ticks and suids. However, there was no direct evidence for the presence of Ornithodoros ticks or wild suids in our study area which is intensively cultivated. This would imply that the novel CVR variants may have evolved outside our study area. It is also possible that the sequence is intrinsically rapidly evolving due to the repetitive nature of some sections of the gene encoding the CVR.

The ASFV isolates in this study were separated into three clusters, based on CVR polymorphisms that were geographically distinct. The first cluster included most of the isolates analyzed in this study (66.6%) that were identical to ASFV isolates associated with outbreaks in earlier studies across Kenya and Uganda which belonged to CVR subgroup XXIV [12, 13, 25]. The second and third clusters were characterized by novel variations within the same region (between positions 185 and 189 of the nucleotide alignment) indicating that this region was more variable within Kenyan and Eastern Uganda isolates than previously realized. The rapidly evolving substitutions within this locus were revealed to be non-synonymous upon translation, identifying an additional variant within CVR subgroup XXIV and a novel CVR subgroup XXIVa which could indicate that the virus is under selection pressure. Interestingly, the CVR subgroup XXIVa contains the tetrameric repeat ‘F’ as one of its tetrameric repeats according to the accepted coding system [16]; a feature that is more typically associated with European, Brazilian, and Caribbean isolates, within *p72* genotype I. This study is the first to reveal the presence of this variant in East Africa. The novel subgroup XXIVa and the novel CVR subgroup XXIV variant were restricted to Western Kenya, specifically Busia County among the regions sampled in-depth. The Ugandan isolates evaluated in this study also clustered together with the novel CVR subgroup XXIV variant. The similarity between Kenyan and Ugandan isolates within the narrow time window of this study further emphasizes the transboundary nature of the disease. Even though all the 2011–2013 CVR nucleotide sequences contained unique variations in positions 317 and 318 of the alignment, the translated sequences indicated that these mutations were synonymous and therefore had no effect on the amino acid composition of the isolates within the *B602L* ORF.

Although the generally assumed pathway of ASFV transmission in Kenya is from West to East, the phylogeographic analyses of the CVR locus is also consistent with the possibility that the ASFV isolate belonging to CVR subgroup XXIV could have originated from Kisauni in Mombasa County in 2011. The virus could have then been transmitted to Central Kenya spreading across Kiambu, Machakos, Athi River, Nairobi, and part of Rift valley in Nakuru before reaching Western Kenya in Busia County from whence it was transmitted back to Central Kenya within 2012 and 2013. This represents an interesting alternative scenario to the generally accepted hypothesis of West to East ASFV migration. The two novel variants detected only within Busia County appear to have evolved from the ASFV CVR subgroup XXIV. However, the presence of the novel ASFV CVR subgroup XXIV variant in Kakamega County, Alupe Uganda, and Nyadorera indicated that the new variant was probably persistent and frequently transmitted, since it was identified in nine isolates during 2013 and was transmitted to other parts of western Kenya. By contrast the novel CVR subgroup XXIVa appeared less transmissible since it was only detected in Busia County and was associated with only three isolates despite the fact that it was first isolated in 2012.

The information gathered in this study could be used to inform strategies for future epidemiological surveillance. Sampling of blood and tissues was primarily informative only during suspected ASFV outbreaks. Random sampling in endemic areas in the absence of reported cases will require a different approach to yield useful data on ASFV prevalence in carrier pigs that are typically seronegative and PCR negative in blood, although viral genomic DNA can sometimes be detected in other tissues following post-mortem [14]. The study suggests that transmission of ASFV could be reduced by improved hygiene and biosecurity at informal slaughter slabs given the high prevalence detected in tissues after slaughter. The close genetic similarity between Kenyan and Ugandan ASFV isolates within a defined time frame confirms the suspected transboundary nature of the virus, highlighting the urgent requirement for intergovernmental cooperation in controlling the spread of ASFV within East Africa. In conclusion, the study provides further evidence that ASFV is endemic in Kenya and Eastern Uganda and that the *p72* genotype IX is associated with all the clinical outbreaks between September 2011 and December 2013. The multiple variations observed within the CVR locus and the detection of two novel CVR subgroups, coupled with the output from the phylogeographic analyses indicate rapid evolution and transmission of ASFV in western Kenya as exemplified by our in-depth data derived from the *B602L* locus within our study region centered in Busia County and adjacent areas of Uganda.

## Electronic supplementary material

Below is the link to the electronic supplementary material.

Supplementary Fig. 1 Phylogenetic tree based on the C-terminal end of the *p72* protein comparing the Kenyan and Eastern Uganda ASFV isolates collected in this study (●) between 2011 and 2013 with other African swine fever virus isolates belonging to ASFV genotypes IX and X. A total of 91 distinct taxa were used to infer a Minimum Evolution tree and the percentage of replicate trees in which the associated taxa clustered together in a bootstrap analysis (1000 replicates) are shown adjacent to the branches. The tree is drawn to scale; with branch lengths represented using the same units as the evolutionary distances used to infer the phylogenetic tree. Supplementary material 1 (DOCX 51 kb)

Supplementary Fig. 2 Phylogenetic tree highlighting genetic conservation within the *E183L* gene within the Kenyan and Eastern Uganda ASFV isolates in comparison to reference nucleotide sequences obtained from GenBank. Supplementary material 2 (DOCX 34 kb)

Supplementary Fig. 3 Amino acid sequences translated using SeqPublish highlighting synonymous substitutions within the thymidine kinase gene in the ASFV isolates obtained from Central Kenya. Supplementary material 3 (DOCX 222 kb)

Supplementary Table 1 Summary of the data obtained from ASFV isolates selected for genotyping in this study and the respective GenBank accession numbers. Supplementary material 4 (DOCX 19 kb)
