# Supplementary figures and images for: Multi-locus sequence typing of African swine fever viruses from endemic regions of Kenya and Eastern Uganda (2011–2013) reveals rapid B602L central variable region evolution
Source: Virus Genes. 2017 Nov 15;54(1):111–23. doi: 10.1007/s11262-017-1521-4 (PMC5847163; doi:10.1007/s11262-017-1521-4)

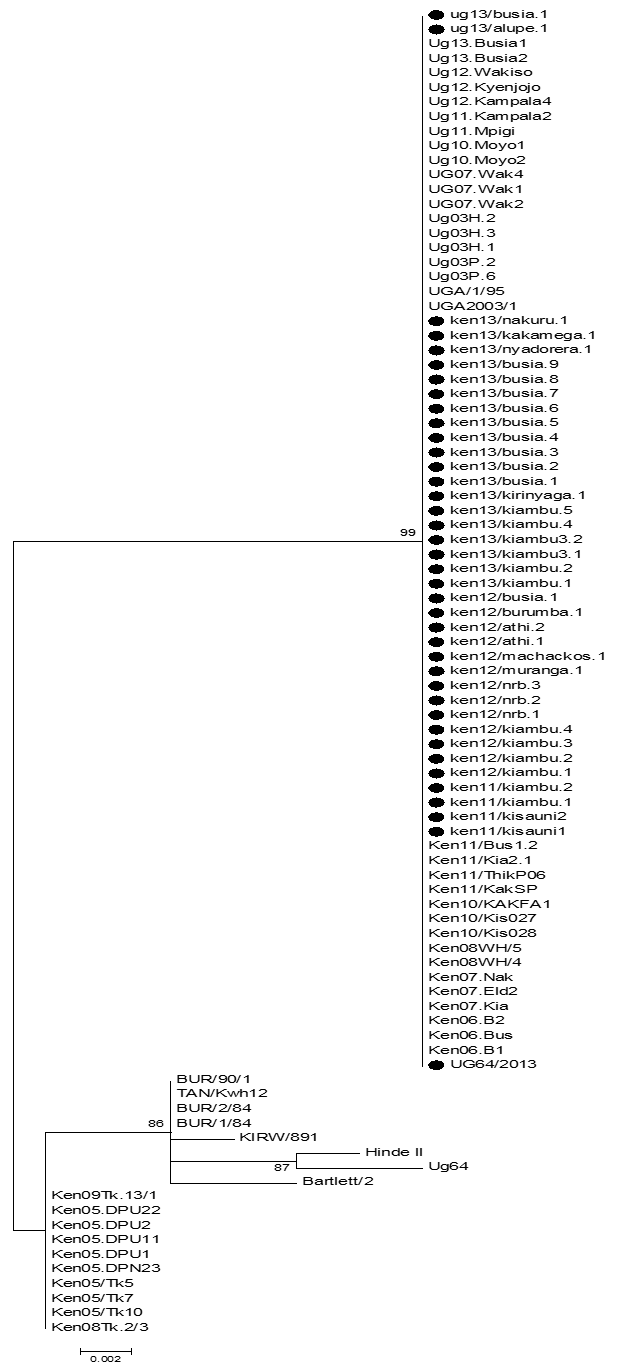


Genotype X

Genotype IX

Supplement: Supplementary file 1 — Supplementary Fig. 1 Phylogenetic tree based on the C-terminal end of the p72 protein comparing the Kenyan and Eastern Uganda ASFV isolates collected in this study (●) between 2011 and 2013 with other African swine fever virus isolates belonging to ASFV genotypes IX and X. A total of 91 distinct taxa were used to infer a Minimum Evolution tree and the percentage of replicate trees in which the associated taxa clustered together in a bootstrap analysis (1000 replicates) are shown adjacent to the branches. The tree is drawn to scale; with branch lengths represented using the same units as the evolutionary distances used to infer the phylogenetic tree. Supplementary material 1 (DOCX 51 kb) [file 11262_2017_1521_MOESM1_ESM.docx]

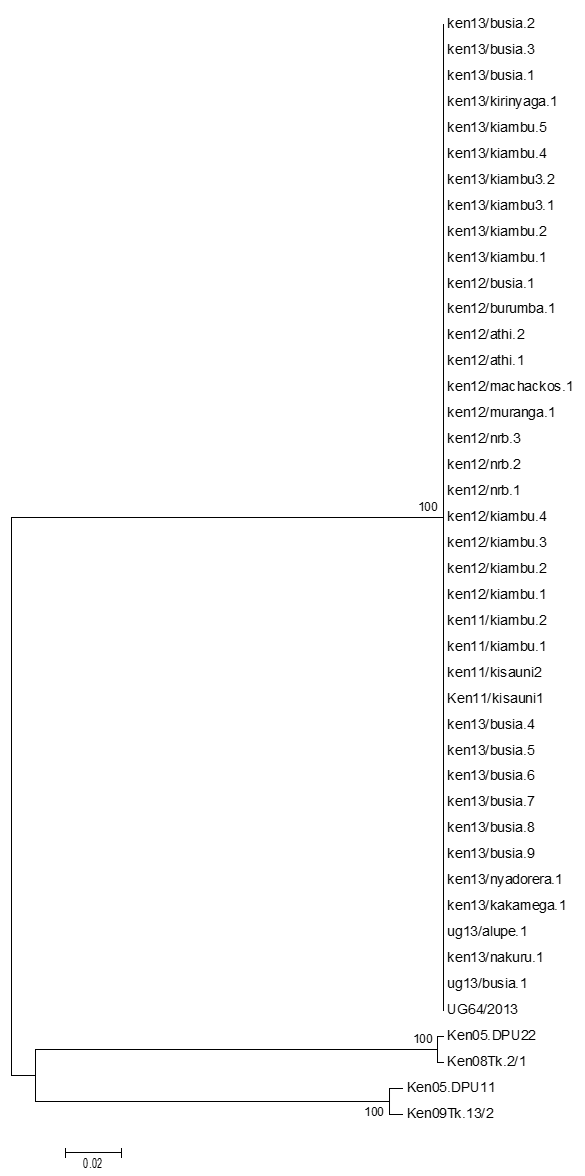


Reference sequences

Supplement: Supplementary file 2 — Supplementary Fig. 2 Phylogenetic tree highlighting genetic conservation within the E183L gene within the Kenyan and Eastern Uganda ASFV isolates in comparison to reference nucleotide sequences obtained from GenBank. Supplementary material 2 (DOCX 34 kb) [file 11262_2017_1521_MOESM2_ESM.docx]

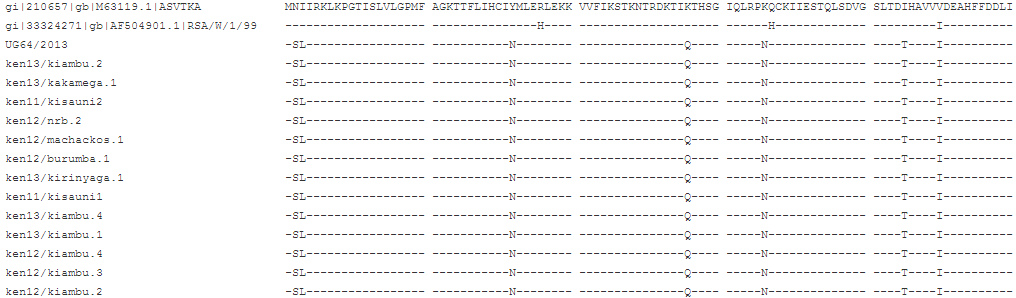


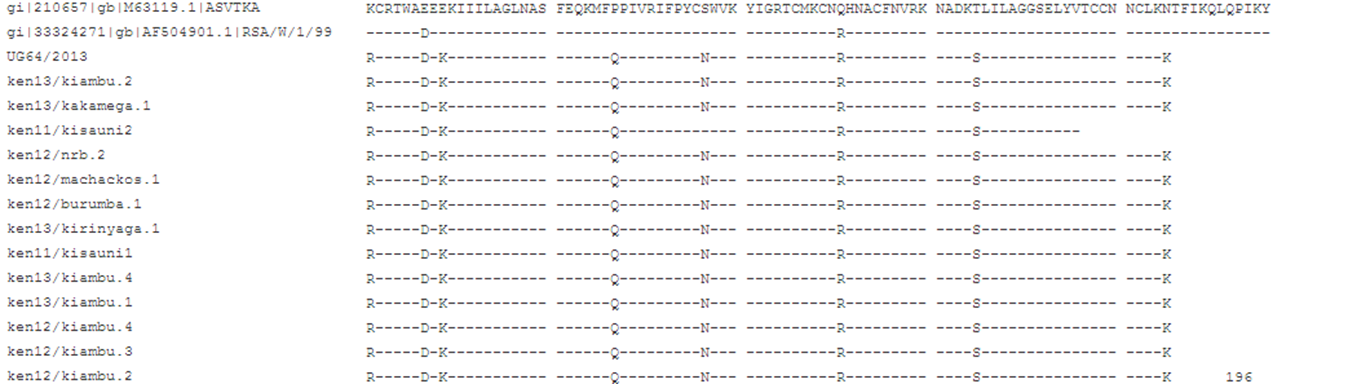

Supplement: Supplementary file 3 — Supplementary Fig. 3 Amino acid sequences translated using SeqPublish highlighting synonymous substitutions within the thymidine kinase gene in the ASFV isolates obtained from Central Kenya. Supplementary material 3 (DOCX 222 kb) [file 11262_2017_1521_MOESM3_ESM.docx]
