# Supplementary material for: Multi-locus sequence typing of African swine fever viruses from endemic regions of Kenya and Eastern Uganda (2011–2013) reveals rapid B602L central variable region evolution
Source: Virus Genes. 2017 Nov 15;54(1):111–23. doi: 10.1007/s11262-017-1521-4 (PMC5847163; doi:10.1007/s11262-017-1521-4)
Supplement: Supplementary file 4 — Supplementary Table 1 Summary of the data obtained from ASFV isolates selected for genotyping in this study and the respective GenBank accession numbers. Supplementary material 4 (DOCX 19 kb) [file 11262_2017_1521_MOESM4_ESM.docx]

| **Sequence ID** | **p72 genotype** | **GenBank accession No.** | **p54 genotype** | **GenBank accession No.** | **CVR subgroup** | **GenBank accession No.** | **Thymidine kinase Genbank accession No.** |
| --- | --- | --- | --- | --- | --- | --- | --- |
| ken11/Kisauni1 | IX | KJ626191 | IX | KJ626193 | XXIV | KJ626195 | KU199307 |
| ken11/Kisauni2 | IX | KJ626192 | IX | KJ626194 | XXIV | KJ626196 | KU199302 |
| ken11/kiambu.1 | IX | KM000132 | IX | KM000168 | XXIV | KM000204 |  |
| ken11/kiambu.2 | IX | KM000133 | IX | KM000169 | XXIV | KM000205 |  |
| ken12/kiambu.1 | IX | KM000134 | IX | KM000170 | XXIV | KM000206 |  |
| ken12/kiambu.2 | IX | KM000135 | IX | KM000171 | XXIV | KM000207 | KU199312 |
| ken12/kiambu.3 | IX | KM000136 | IX | KM000172 | XXIV | KM000208 | KU199311 |
| ken12/kiambu.4 | IX | KM000137 | IX | KM000173 | XXIV | KM000209 | KU199310 |
| ken12/nrb.1 | IX | KM000138 | IX | KM000174 | XXIV | KM000210 |  |
| ken12/nrb.2 | IX | KM000139 | IX | KM000175 | XXIV | KM000211 | KU199303 |
| ken12/nrb.3 | IX | KM000140 | IX | KM000176 | XXIV | KM000212 |  |
| ken12/muranga.1 | IX | KM000141 | IX | KM000177 | XXIV | KM000213 |  |
| ken12/machakos.1 | IX | KM000142 | IX | KM000178 | XXIV | KM000214 | KU199304 |
| ken12/athi.1 | IX | KM000143 | IX | KM000179 | XXIV | KM000215 |  |
| ken12/athi.2 | IX | KM000144 | IX | KM000180 | XXIV | KM000216 |  |
|  |  |  |  |  |  |  |  |
| **Sequence ID** | **p72 genotype** | **GenBank accession No.** | **p54 genotype** | **GenBank accession No.** | **CVR subgroup** | **GenBank accession No.** | **Thymidine kinase Genbank accession No.** |
| ken12/burumba.1 | IX | KM000145 | IX | KM000181 | XXIV | KM000217 | KU199305 |
| ken12/busia.1 | IX | KM000146 | IX | KM000182 | XXIVa | KM000218 |  |
| ken13/kiambu.1 | IX | KM000147 | IX | KM000183 | XXIV | KM000219 | KU199309 |
| ken13/kiambu.2 | IX | KM000148 | IX | KM000184 | XXIV | KM000220 | KU199300 |
| ken13/kiambu3.1 | IX | KM000149 | IX | KM000185 | XXIV | KM000221 |  |
| ken13/kiambu3.2 | IX | KM000150 | IX | KM000186 | XXIV | KM000222 |  |
| ken13/kiambu.4 | IX | KM000151 | IX | KM000187 | XXIV | KM000223 | KU199308 |
| ken13/kiambu.5 | IX | KM000152 | IX | KM00188 | XXIV | KM000224 |  |
| ken13/kirinyaga.1 | IX | KM000153 | IX | KM000189 | XXIV | KM000225 | KU199306 |
| ken13/busia.1 | IX | KM000154 | IX | KM000190 | XXIV | KM000226 |  |
| ken13/busia.2 | IX | KM000155 | IX | KM000191 | XXIVa | KM000227 |  |
| ken13/busia.3 | IX | KM000156 | IX | KM000192 | XXIVa | KM000228 |  |
| ken13/busia.4 | IX | KM000157 | IX | KM000193 | XXIV | KM000229 |  |
| ken13/busia.5 | IX | KM000158 | IX | KM000194 | XXIV | KM000230 |  |
| ken13/busia.6 | IX | KM000159 | IX | KM000195 | XXIV | KM000231 |  |
| ken13/busia.7 | IX | KM000160 | IX | KM000196 | XXIV | KM000232 |  |
| ken13/busia.8 | IX | KM000161 | IX | KM000197 | XXIV | KM000233 |  |
| **Sequence ID** | **p72 genotype** | **GenBank accession No.** | **p54 genotype** | **GenBank accession No.** | **CVR subgroup** | **GenBank accession No.** | **Thymidine kinase Genbank accession No.** |
| ken13/busia.9 | IX | KM000162 | IX | KM000198 | XXIV | KM000234 |  |
| ken13/nyadorera.1 | IX | KM000163 | IX | KM000199 | XXIV | KM000235 |  |
| ken13/kakamega.1 | IX | KM000164 | IX | KM000200 | XXIV | KM000236 | KU199301 |
| ug13/alupe.1 | IX | KM000165 | IX | KM000201 | XXIV | KM000237 |  |
| ken13/nakuru.1 | IX | KM000166 | IX | KM000202 | XXIV | KM000238 |  |
| ug13/busia.1 | IX | KM000167 | IX | KM000203 | XXIV | KM000239 |  |
| Ug64/2013 | IX | KX776419 | IX | KX776420 | XXIV | KX776421 | KU199299 |
